# The perceptions of turkish immigrants towards discrimination and racism during the last ten years: An empirical analysis of quantitative survey data

**DOI:** 10.1192/j.eurpsy.2021.855

**Published:** 2021-08-13

**Authors:** E.D. Cindik Herbrüggen

**Affiliations:** Psychosomatik/psychotherapie, Neuro-Psychiatric Center Riem (Neuro-Psychiatrisches Zentrum Riem), München, Germany

**Keywords:** perceived discrimination, discrimination, Turkish immigrants

## Abstract

**Introduction:**

Turkish people immigrated to Germany initially as guest workers since the 1950s. Even though some Turkish immigrants resist to integrate culturally in order to preserve their traditions, those who immigrated during the last ten years considered themselves as part of the German society. It is hypothesized that Turkish immigrants experience more discrimination in the labor market and in education life since the early years of immigration. In addition, they feel more discriminated in comparison to immigrants from other nations.

**Objectives:**

This paper aims to investigate the perceptions of Turkish immigrants towards discrimination in their daily lives. Besides, the relationship between being discriminated and having mental disorders is investigated.

**Methods:**

125 participants aged between 18 to 70 years were surveyed through a specific questionnaire. Moreover, face to face interviews were held to gain more insights into participants perception of discrimination.The relationship between being discriminated and having mental disorders was analyzed.

**Results:**

The preliminary findings illustrate that while the perception of the elderly Turkish immigrants towards discrimination is higher, the younger immigrants feel more integrated and in the society. Turkish immigrants mostly experience discrimination in education life (27.3 %), labor market (30. 8.%), and while house seeking (50.3 %). 38.5 % participants also indicate that they felt very depressed and stressful after they experienced discrimination during the last ten years. Turkish immigrants (58 %) feel more discriminated in comparison to other immigrants.

**Conclusions:**

The results of the study demonstrate that there is a relationship between having mental disorders and being discriminated in daily life as an immigrant.

